# Mr. Tully on the Plague of Malta, Gozo, Corfu, Cephalonia, &c. &c.

**Published:** 1821-12-01

**Authors:** 


I. *The History of Plague, as it has lately appeared in the Islands of Malta, Gozo, Corfu, Cephalonia, &c. detailing important Facts, illustrative of the Specific Contagion of that Disease, with particulars of the Means adopted for its Eradication.* By J. D. TULLY, Esq. Surgeon to the Forces, Member of the Ionian Academy, late Inspector of Quarantine, and President of the Board of Health of the Ionian Islands. One vol. 8vo, pp. 292. London, 1821.

II. *Researches into the Laws and Phenomena of Pestilence; including a Medical Sketch and Review of the Plague of London, in* 1665: *and Remarks on Quarantine. With an Appendix: containing Extracts and Observations relative to the Plagues of Morocco, Malta, Nova, and Corfu: being the Subject of the Anniversary Oration, delivered before the Medical Society of London, in the Spring of* 1820, *and published at their Request.* By THOMAS HANCOCK, M. D. Licentiate of the Royal College of Physicians, and Physician to the City and Finsbury Dispensaries. One vol. 8vo. pp. 379. London, 1821.

---

"While vapours blown by Auster's sultry breath,
"Pregnant with *plagues*, and shedding seeds of death,
"Beneath the rage of burning Sirius rise."—ILIAD. V.

---

"I have sent among you the *pestilence* after the manner of Egypt; and I have made the stink of your camps to come up into your nostrils."
AMOS.

---

FEW nations, at one or other period of their history, have escaped the ravages of plague; and unfortunately the inhabitants of those countries in which it seems to have fixed its head quarters, are so strongly impressed with a conviction of its irresistible power that they succumb before it, without a struggle, regarding its appearance among them as a direct visitation from Heaven, and considering any measures of prevention or resistance as little less than impiety! What can be more reasonable than that, among such people, the fomites of the disease are never extinct, though various circumstances may render them at one time apparently epidemic, and at another nearly dormant. These vicissitudes have furnished the anti-contagionists with a pretext (not a very plausible one either) for confounding PLAGUE with those endemics which rise and fall at particular seasons of the year, and depend, as far as we have yet been able to ascertain, on certain conditions of air and earth, not cognizable indeed by the senses, but too evident from their deleterious effects on the human constitution.

Happily for mankind, the concurrent testimony of ages has induced a general belief that plague arises from a specific contagion—that it is communicated by contact (or exceedingly close proximity) alone—and that seclusion and separation are the only means of checking, and ultimately exterminating its power.

Fanciful theorists, indeed, have started up in all ages, to deny the contagion of plague, and generally have forfeited their own, and thousands of other lives, in consequence of their temerity. Thus, during the plague at Marseilles, in 1720, the physicians *in Paris* determined that it was not contagious, and their delegates at Marseilles acting conformably to this opinion, *sixty thousand* people fell victims to the disease in the space of seven months. A similar prepossession induced the faculty in Sicily to declare the plague which ravaged Messina in 1743, not to be contagious, and forty-three thousand lives were lost. The doctrine of non-contagion, however, being in these instances, so immediately refuted by facts, these cases can only be considered as insulated exceptions to the general belief in contagion. Those, too, who have had most experience in plague, are those who most confidently believe it contagious; while the non-contagionists are generally speculative closet physicians, who have seen little or nothing of the disease. Thus Russel, Howard, Fra Luigi de Pavia, Raymond of Marseilles, Giovanelli of Leghorn, Sir James M'Grigor, Sir Brooke Faulkner, and, in fact, all British physicians, who have had any experience of plague, assert its contagious character, while Dr. M'Lean, determined on its being non-contagious long before he ever visited those countries where it is prevalent, travelled with his eyes blinded to the most obvious facts, (for surely a brace of pestilential buboes in his own groins were argumenta ad hominem,) and returned with all that he could scrape together, on one side of the question, but carefully slurring over all that presented on the other. As an example of this sort of *fair* investigation, we shall here bring forward an important fact stated by Mr. Tully, and personally witnessed by Dr. Greaves, Inspector of Military Hospitals, and Superintendant of Quarantine in the Island of Malta.

An extensive building at the extremity of Strada Vescoso, in Valetta, had, for years, been occupied as a military hospital, and was only separated from Dr. Greaves's house by a narrow lane, not more than ten feet in breadth. During the prevalence of plague, this hospital was occupied by the sick of one of the battalions quartered in the town, admitting the ordinary proportion of the common diseases incidental to the climate, the season, and military life. The under apartments of the hospital were partitioned into several distinct habitations, dry and comfortable; while the site of the building itself was allowed by all to be one of the healthiest in Valetta. The several entrances to these apartments were lofty and spacious, and each dwelling gave an asylum to Maltese—in the proportion of about seven to each apartment. Plague, which was making havoc in the neighbourhood, at length penetrated these apartments, seven in number, four of which were, in a few days, completely depopulated; while in the remaining three, two individuals only, of each family, escaped. Independently of the ravages that were committing below, many of the families in the immediate vicinity were attacked, "contagion extending indiscriminately to the upper as well as the lower apartments of these dwellings." The inmates of the hospital were closely shut in, every avenue to intercourse between them and the inhabitants of the town was securely guarded, and all communication effectually cut off. What was the consequence? "While plague was running its destructive course in every angle, every individual within the walls of the hospital continued perfectly free from danger, and this exemption from plague continued throughout the disease." P. 250.

If the non-contagionists remark that in marshy countries, and during the prevalence of miasmal fevers, the lower ranges of buildings sometimes suffer, while the upper are free from disease, it may be answered that to talk of marsh miasmata at Malta is to talk nonsense. Remittent and intermittent fevers are there almost unknown, and there is but one single spot on the whole island that ever offered any thing like a marsh, and little or no vestige of it now exists.

"But the most extraordinary part of this statement is yet untold. I have been assured by Dr. Greaves, that the circumstances here narrated, relative to the security which seclusion afforded to the sick in the hospital in Strada Vescoso, was communicated by him (and no doubt with the same candour and view to truth with which he was pleased to communicate it to me,) to Dr. Maclean, the learned author of "*Results* of an Investigation respecting epidemic and pestilential Diseases," who was then at Malta. Dr. Greaves was not satisfied with stating the fact, but he conducted Dr. Maclean, as he did myself, to the very spot, clearly pointing out every circumstance which, in his opinion, was well calculated to throw light upon the subject of Dr. Maclean's investigation. Now, with all deference to Dr. Maclean, I cannot help remarking, that his taking no notice whatever, in his publication, of this truly important information, does not argue much in favour of the candour with which he either treated the public, or conducted his investigation; for, however the fact might have militated against his favourite doctrine, it behoved him to state it. As he has not done so, we certainly are warranted in concluding that, having long previously established in his own mind that contagion was a non-entity, the information he required at Malta was of that nature which he considered it better to consign to oblivion than to submit to the test of inquiry. Facts are stubborn things, and I candidly confess that the one just recorded is reluctantly brought forward; but were I to act otherwise, I should be as culpable as Dr. Maclean, who, in presenting to the public his "Researches," withheld it." 260.

These "*Researches*" met their well-deserved fate in the Plague Committee of the House of Commons, and are now doomed to the mouldering shelves of the booksellers, or such other purposes as they may be fit for.

While we agree with Mr. Tully in denying that the contagion of plague is diffusible in the atmosphere to a great distance, as maintained by Dr. Calvert, we cannot coincide in the opposite opinion, that climate, seasons, and the extremes of heat and cold, have no influence whatever on the rise and fall of this dreadful scourge. We all know that caravans are constantly conveying susceptible goods from Damascus and Aleppo to Bussorah, from whence they are shipped for the East Indies; yet it does not appear that the plague ever reached Guzerat, Surat, Bombay, or any other part of the East Indies. Mr. Tully himself thinks—"it is not improbable that this exemption may be owing to the high atmospheric temperature in those latitudes being suffered to destroy the contagion of plague—a temperature unknown in those countries which are the constant seat of this malady." If Mr. Tully had resided in the East, and also in Malta, Gibraltar, and other Mediterranean ports, where plague has visited, he would have known that few years pass in these latter places without a complete tropical temperature for many weeks, each hot season. But Dr. Russel has shewn that, speaking generally, the plague rises and falls at certain *seasons*, in Aleppo, and therefore while we grant that the cause of the disease is *always* contagion, we have no doubt that the activity of the poison is increased, diminished—perhaps sometimes almost annihilated by climate, seasons, and other atmospherical or terrestrial influences, with the nature of which we are not yet sufficiently acquainted. Why should not this be the case with plague as well as with small pox?

The opportunities which have been, unfortunately, afforded to British subjects, of late years, to ascertain the true nature of plague, have been so numerous, that all speculations on the subject can be brought to the test of practical inquiry. The author, whose work we are reviewing, had, himself, nearly six years practice, as chief of the health department in those countries so often visited by plague, and consequently his observations are entitled to great attention, however some people may differ with him in certain matters of opinion or doctrine.

The 2d chapter of the work before us is on the plague in Malta, as it occurred at different periods, from the year 1592 to 1813. Into an account of the earlier plagues it would be improper to enter here, and the recent visitation is sufficiently fresh in the memory of the profession and public at large. It has been too justly observed by a modern philosopher, that "he who has never suffered extreme adversity, knows not the full extent of his own depravation." It is on such awful occasions as these, when pestilence is abroad, and death mowing down his victims, that the dark as well as the bright side of human nature becomes particularly developed!

"Alarm, (says Mr. Tully, speaking of Malta in 1813,) every where prevailed. Self-preservation was the only acknowledged law, and all alike dreaded their fellow creatures.

"Dependents, friends, relations, Love himself,
"Savaged by woe, forgot the tender tie,
"The sweet engagements of the human heart."

Few have been so successful (we hope we cannot properly say—happy) in delineating, with disgusting fidelity, these dark traits of the human mind, as Lord Byron. We envy not his Lordship's feelings—we admire not his taste—we approve not his philosophy, in this line of conduct. Were these depravities of our nature held up for detestation, there would be some excuse; but when they are rendered familiar, or even attractive, by the splendour of poetry, they are calculated to debase the human soul to this frightful point, rather than elevate it to a nobler aim and destiny.

The decline of the plague of 1813 at Malta is attributed, and we should suppose justly, to the energetic measures of Government—particularly of Sir Thomas Maitland, whose arrival on the island formed "a particular æra in the history of the Plague of Malta;" the disease being every where "arrested by the decided and energetic system established and enforced by His Excellency." This conquest over the annihilating monster was not an easy one—"Casal Curmi boldly contesting, inch by inch, every effort which was made for the destruction of the disease." For although the necessity of the restrictive system was fully recognized, yet evasion, on the part of those who are ever ready to profit by the general consternation around them, occurred, and thus a public calamity was converted into the means of furthering private and nefarious ends! By the early plunder and concealment of infected goods, disease was kept alive in many parts of Malta, although the wretches thus offending against the community fell daily sacrifices to the wrongs they had committed. On a sudden, however, "that arm was raised which, under Providence, was destined to crush the desolating foe."

In the Casal Curmi the plague for a long time bade defiance to every exertion, and thus favoured the idea of its dependence on atmospherical constitution. But His Excellency had now recourse to the novel and extraordinary plan of converting a populous country town into a lazaretto, insulating the inhabitants by double walls, and cordon over cordon. In all times, ancient and modern, the circumvallation of a *common* enemy has been found difficult; but to effectually circumscribe such an enemy as the plague, ravaging a large town, was reserved for Governor Maitland. So completely was this work performed, that all danger was speedily at an end —retreat was rendered impossible—the disease was limited to the town—and, although the germs of plague were not entirely extinguished for a considerable time within *this* casal, yet there was not a single instance of any of the troops, forming the cordons around it, having suffered while on that service. Mr. Tully may well say—"if this is not characteristic of specific contagions, we confess ourselves at a loss for an appropriate term to apply to this disease." Certainly if the epidemic (as it has been called) sprung from a peculiar constitution of air, at the time, it is difficult to conceive how the circumvallating corps escaped—we mean it is difficult to every one but an anti-contagionist.

" For all is easy to the *œtherial* kind."—ODYSSEY.

So soon as the disease was confined within the walls of CASAL CURMI, all anxiety respecting further danger ceased; " and although the plague was in the very heart of the country, it produced no more dread or alarm than if no such disease existed. No precautions were thought of *without* the cordons." 64.

" It is worthy of remark, that whilst many of the inhabitants of Valetta and of some of the casals, were, after the first alarm had subsided, wrapped in fatal security, unapprehensive of any dangerous result, the inhabitants of the three cities Vittoriosa, Cospicua, and Senglea, were more alive to the true consequences of the evil, and under this alarm immediately set about concerting measures for their own safety.

" The city of Valetta was, at the time alluded to, in open communication with all the other parts of the island, of course, excepting the lazaretto. The inhabitants of the three cities abovementioned, jealous of their safety, and impressed with the conviction that contagion was abroad, had recourse to the bold step of cutting off all communication with the capital, and for a time, even government officers, under the seal of authority, were peremptorily denied a landing by the populace of Senglea." 67.

This bold step, whether legal or illegal, saved the inhabitants who resorted to it. The population of these three cities exceeded that of the capital. Our author concludes then, that the late plague of Malta offers in its progress the most irresistible proofs of the specific contagion of the disease, and, in pursuing it to its close, we find its history still true to the same principle.

The 3d chapter discloses some curious information relative to the Island of Gozo. The inhabitants of this little spot, aided by the exertions of Government, took the most rigorous precautions to cut off all communication between Valetta and themselves, as soon as the plague broke out in the former place. In consequence of this, they " enjoyed an uninterrupted good health," during the whole period of the disease in Malta. When all restrictions were taken off in the latter place, the communication opened, but the consequences were soon seen. A man of the name of Galen, an inhabitant of CASAL CURMI, secreted a box of infected clothes, previously to the circumvallation of the above ill-fated town. When the pratique was taken, he recovered the box, and passed over to Gozo, of which he was a native. Two or three days after his arrival there, viz. on the 22d of

February, 1814, he died suddenly at his own house, in Casal Caccia, and, as there was no suspicion at the time, his corpse was carried to the parish church, and the usual funeral ceremonies performed. On the 28th of the same month, Rosa, his daughter, was taken ill, and being carried to the hospital, she died in a few hours of the plague. The alarm was now given, and in a few days several inhabitants of Casal Caccia died, among whom was the priest who assisted in Galen's burial. The infected part of Gozo was now immediately circumvallated, and the disease thus prevented from spreading, while the proper processes of purification soon put an end to the disease within the lines. Out of about 15,000 souls 96 died on this island. Among the latter was Dr. M'Adam, physician to the forces—a non-contagionist. It is a remarkable fact, Mr. Tully observes, that, of the many British medical officers employed in the recent plagues that have occurred under the British flag, within the Mediterranean, "the disbelievers in the long-established doctrines of the specific contagion of the disease were *alone* its victims."

The 4th chapter, on the plague of 1813, 1814, on the banks of the Lepanto, and in Albania, is not very interesting or instructive, dealing too much in generalities, and too little in specific facts. We shall therefore pass it over intact.

The 5th chapter, on the plague at Corfu, is the longest, most elaborate, and most circumstantial, in the volume. It is apparently that one which, in Mr. Tully's opinion, contains the most decisive evidence of the contagious nature of plague, and of the range of the contagion being limited to actual contact. Yet to us it is exceedingly unsatisfactory, and we doubt not that it will furnish the anti-contagionists with ample materials for opposition to our author's doctrines and quarantine regulations. The tenor of this chapter, and many of the facts almost irresistibly impressed us with the idea that a quarantine officer sees nothing but contagion, and consequently that his statements must be taken *cum grano salis.* Another idea forced upon us by many of the facts and circumstances is, that the disease in Corfu was not plague, but one of those aggravated endemics, which become actually epidemic, and contagious at the same time, from a combination of causes which it is exceedingly difficult to unravel. We cannot pretend to offer any analytical view of this chapter, as it would require a larger space than we can afford for the whole article. We can only extract a few prominent particulars.

It was in the little village of Marathia, in the district of

Leftimo, on the Island of Corfu, that plague was first discovered in December, 1815. It was found, on investigation, however, that a fever had broken out amongst this little community, more than a month previously, which had assumed a most malignant form, thirteen out of a population of fifty having died. The cause which superstition had assigned for this scourge, was the perturbed spirit of a man who had been murdered in the neighbourhood of the village some months before. To make every atonement to the angry spirit, church-offerings, prayers, and processions were tried in vain. The more probable causes, and more physical at the same time, will become obvious in the following extract:—

"The village of Marathia is situated on a little eminence or ridge running along the southern extremity of the island, and nearly dividing it, looking towards both seas; on either side of this ridge, the ground runs into a flat, and stagnant pools and marshes every where present themselves. During the autumnal months, the remittent fever had been most destructive in this district, very few having escaped its attack. The season had been extremely mild, the rains set in earlier than usual, heavy but partial, and were followed by a long drought and heat unnatural for the advanced season of the year, the thermometer being seldom below sixty-six, with a constant *sirocco*, or south-east wind." 90.

Mr. Tully ingenuously confesses that, when he reflected on the topography of the place, the nature of the disease, the poverty "amounting almost to absolute want," of the inhabitants, the natural insalubrity of the whole district, the inland residence of the peasantry, surrounded by marshes, and "wholly unconnected with commerce," he and those who acted with him were impressed with a conviction, that "the disease before them was the offspring of the soil."

"*Conceiving* (however) that it was not only of a malignant, but also of a contagious nature," our author determined to adopt the strictest measures of precaution. Accordingly a board of health was constituted, two professional gentlemen were dispatched to Marathia, and the infected village was circumvallated as Casal Curmi at Malta. Notwithstanding these precautions, however, the disease was *disseminated*—at least dissemination was inferred, as "a disease similar to that with which the inhabitants of Marathia were afflicted, had broke out in the neighbouring villages." The line of contagion or contact was traced to a knot of *papâs*, or clergymen, from various villages, who had, previous to the circumvallation, assembled at Marathia, to assist the inhabitants in a religious ceremony for appeasing the angry spirit, the supposed cause of all the mischief. The medical inquisition accordingly investigated this new route, and "disease was traced, step by step, to the residence of every individual who had assisted in the ceremony at Marathia, and from them again to their friends, &c." We need hardly inform our author that these clergymen might have become affected with febrific miasmata at Marathia, as readily as with contagion; and among such miserable, dirty, and superstitious wretches, there can be little difficulty in conceiving that the disease would continue to be propagated from individual to individual. A report to Government having been drawn up, in conformity with the above views, the quarantine regulations were put in still more rigorous execution, and an additional cordon of troops was placed around the enemy. "The erection of barriers followed, and thus all communication was cut off." Well! what was the result of all this? why that, "notwithstand- all these precautionary measures, disease was every day breaking forth"—and that too, in villages *without* the cordons! It is true that Mr. Tully attributes all these disappointments to want of integrity in the *primates* and violation by stealth of quarantine laws; but we confess that his statements are far from satisfying *us*, who are decided believers in the contagious nature of plague. We shall, however, extract one of the most circumstantial facts which Mr. Tully has adduced, in order that we may not be considered as hiding away contagious matters, like the *papás* of Corfu. Speaking of the Herculean force of the disease, and its power of eluding all their vigilance, he details an incident that occurred at the populous village of Perivoli.

"That village had absolutely been forty days exempt from disease, expurgated, and the inhabitants were on the point of being admitted to free pratique amongst themselves, when a report was transmitted to me by Assistant-Surgeon Muir, that a case of asus - picious nature had at that moment attracted his attention. I proceeded without a moment's loss of time to Perivoli, where I was conducted to the residence of a lay brother, whom I found labouring under every symptom of plague in its most aggravated form. He was leaning against his door, and with a firm voice answered the several questions I put to him; from which it appeared, and of which he himself was convinced, that his illness was occasioned by his handling some clerical robes that he had taken from a box deposited under the altar of a neighbouring church. The fact was evident; the robes had been sent there to avoid being expurgated, and the clergyman, who had made the deposit, was attacked with, and died of plague a few days after. All this resulted from our examination, and our returns proved that the clergyman who had officiated at the church, had been included in the bills of mortality nearly three months previous. The fact of the robes having been deposited there, was known to many of the friends of the deceased, but only made known to the public authorities on the eve of pratique being granted, as the church in question had been long the residence of the officer commanding the cordon. As it was necessary that the robes should be expurgated, the primates dreaded that it might interfere with the intended pratique; they therefore persuaded the officer commanding, that the effects belonging to the church had been placed there for security long before the breaking out of the plague. Confiding in this assertion, the officer commanding, imprudently, and without reference to the officers of health, yielded to the request, that the lay brother, the only person surviving belonging to the church, should be permitted to expurgate them. The unfortunate man proceeded to fulfil the duty thus imposed upon him by the inhabitants on the morning I saw him; he had not been absent more than an hour, when he returned under an escort to his house. Soon after his arrival he was seized with giddiness, and other symptoms of plague, that led to the suspicion upon which I was called to decide.

"I had scarce terminated my enquiry, and issued orders for the unhappy man's removal to the lazaretto, when walking up the street, I was requested to return, as the man was apparently dying, and in an unfit state for removal; before I reached the door he was no more. Three hours had only elapsed from the time he had quitted his house in perfect health. This was one of the most sudden cases I ever witnessed; yet this man's sufferings did not appear to be by any means severe.

"The unfortunate person, happily, had no family, not even a servant. We were perfectly satisfied that he had had no communication whatever with any individual from the first appearance of disease in the village; and this, more from his habits of retirement than from any dread of disease. By this fortunate circumstance, the malady was checked on the instant; the effects of the deceased, together with the church robes, were sent to the lazaretto, and the house immediately expurgated. No other case of plague occurring in Perivoli, after the lapse of a quarantine of observation, the pratique, which was, by this fatal accident, interrupted, was granted with the fullest security to the country." 107.

We regret that Mr. Tully should not have stated what those "other symptoms of plague" were, thus leaving us in darkness.

During a period of nearly two months, in which upwards of three hundred of both sexes, and all ages, fell victims, our author acknowledges "the hourly disappointments, hopes, and fears, which accompanied them in every stage of their operations." At length one decisive step was taken. They pitched upon an eligible spot of ground on the sea-shore, to which they conducted every infected and every suspected person in the district, amounting to 300 and upwards, where they were guarded and double guarded till the disease ceased—the habitations, &c. which they left, being, of course, expurgated—that is, we suppose, exterminated.

Knowing, as we all do, that every epidemic will have an end, sooner or later, we greatly suspect that this last move to a more healthy situation would have produced the consequences detailed, whatever was the nature of the disease. But we will not "argufy the topic" any farther here, since we believe the point will never be settled by argument.

The *sixth* chapter of the work before us, contains much interesting matter for those employed in quarantine service, but not requiring any particular notice in this place.

*Chap.* VII. No sooner had our author performed 40 days of foul quarantine at Corfu, than he was dispatched with a "select corps of expurgators," to the Island of Cephalonia, where the plague had broken out, and was making alarming progress. The origin of the pest here was, according to Mr. Tully, "early ascertained." The island is of considerable extent, the population being much diminished, and agriculture still more so. On this account, about a tenth of the population annually migrate to the neighbouring continent, to cultivate and reap the harvest of a foreign land. The mode in which the plague was introduced into Cephalonia is thus stated. Shortly after the breaking out of the disease on the opposite coast, (of the true nature of which there is no evidence) Antonia Venturato and some companions, on their return to Cephalonia, found the bodies of two Turks, "who, a few hours previous had died of plague, (of which also there is no evidence,) and their bodies were stripped of every thing that was valuable." This party reached St. Eufemia, in perfect health, and continued so for nine days. It is somewhat remarkable (we had almost said improbable) that the clothes and other articles taken from the bodies of the Turks by Venturato had been kept carefully wrapped up in a great coat, from the moment they were seized, without ever being looked at or examined, till the ninth day, on the evening of which Venturato was taken ill. But granting that this was the case, why should Venturato not have caught the infection, in the first instance, when despoiling the dead, rather than nine days afterwards, when looking over his ill-got plunder? We must confess that this smacks strongly of quarantine. The indisposition of Venturato now excited much alarm in the whole party, (thirteen in number,) as they were aware that the plague was raging at Arta, "and strongly *suspected* that the Turks were sufferers from that disease." What would Sir Gilbert Blane say to this kind of medical logic, or evidence, even although on the side of contagion? The reason why we examine the allegations with rigour is, that we firmly believe in the contagious character of plague; and, on that account, cannot allow suspicious evidence to pass, for the purpose of being held up by the anti-contagionists, as the general grounds on which we build our faith. No. We will not grant them this advantage.

To return to the narrative. Venturato died on the second day of his disease, in strong convulsions. He was examined, after death, by Dr. Metasea, "a physician of talent and reputation," who reported that there were "no external appearances that could lead to any opinion of the nature of the disease." Nine persons soon died of this disease, in the village of Comitato, generally after a few hours' illness. A medical deputation being dispatched to the scene of alarm, the body of Venturato's brother, who also died of the disease, was examined, and petechiæ of a large size were found thereon, as also a carbuncle on the temporal muscle. In several others the same petechial eruptions were observed, and some buboes were found. Mr. Tully having arrived in the vicinity of the village determined on making a personal examination of the state of affairs.

> "Here the scene was melancholy and distressing. In the general terror which was excited, the sick were heaped indiscriminately together, with the whole of their families, in a house selected for their seclusion, at one extremity of the village—where all, without distinction, were placed—the dead, the dying, the infected, and many even free from disease. The state of the latter was of short duration, as there appeared little hope of escape, immured as they were within the walls of danger, and surrounded by a mass of contagion.
>
> "The consequences of these unfortunate measures were an attempt at concealment on the part of all attacked with disease; and, the more effectually to evade discovery, many buried their fathers, mothers, husbands, and children, in their gardens, or within their houses; indeed, such was the state of desperation to which they were reduced, that some were known to have thrown the bodies of the deceased relatives into their cisterns." 163.

Our author took what measures he could in this exigency —directed them to have their houses, streets, and lanes thoroughly swept and cleansed, as a preparatory step, while poultry, cats, and dogs were prohibited from prowling about, and thus conveying infection from place to place. A small house at the water's edge, in the bay of St. Pantaleone, was pitched upon for an hospital, and here the quarantine camps were erected. To this place the sick and suspected were carefully conveyed, and proper quarantine regulations put into execution. After all were removed to this romantic little bay, new cases of plague occurred daily in the encampment, which was no more than might be expected. These, of course, were instantly put under medical treatment, which is here, as it were incidentally, alluded to.

"The whole of them were then placed under medical treatment, which consisted in a gentle purgative, followed by mercurial inunction, proportioned to the age and constitution of each individual. The mercury was persevered in morning and evening, until the system was under its influence; this practice was only resorted to in the cases of the strongly suspected, who of course were received without any symptoms whatever of plague being on them.*

We cannot enter into the long and minute detail of the ways and means taken for the suppression of this dreaded contagion—suffice it to say that they were successful, and appeared to reward the labour and care bestowed upon them.

The plague of Cephalonia offered nothing in its general character different from the disease of Corfu. No recoveries marked its first appearance, death following every attack until the arrival of the quarantine officers, and 59 persons having fallen a sacrifice to the disease. Mr. Tully's experience does not coincide with a very commonly received opinion, that plague varies in its character, from the period of its commencement till its final termination. In the cases which occurred at Leftimo, the last were equally malignant as the first. The same was the case at Cephalonia.

To the question—"how long may the contagion of plague remain in the constitution before the morbid action becomes evident?" our author is positive in stating that the period never extends to twenty days, but is generally much within it.

The 8th chapter of the work presents a very rapid sketch of the late plague at Noia. The manner in which the contagion was first introduced, is unknown. It broke out on the 25th of November, 1815, and continued raging nearly six months. The constituted authorities, despairing of segregating the infected or suspected part of the population, circumvallated the whole town, and drew trenches around

"* The efficacy of these means, with the medical history and treatment of the plague in the Ionian Islands, will be speedily submitted to the profession."

it which were guarded by 1200 men. The inner cordon of troops was only sixty paces from the infected habitations, and no case of the disease appeared among them or outside of the circumvallation, during the whole period of the plague raging within. This was undoubtedly a most decisive proof of the contagion of plague, and of its limited range.

The 9th chapter embraces some general observations arising out of the subject matter of the work, which we shall briefly touch upon before we close this article. The first conclusion which our author draws—a momentous one indeed—is, that the history of the disease, as far as his experience goes, offers no one instance of any person being attacked with the disease "by near approach to, or by breathing the same atmosphere with the sick; whilst every attack, in many hundred cases that came under his own eye, was satisfactorily traced to positive contact with infected persons or things." In all cases where communication was cut off, danger was unknown—the disease offering "respectful regard for all public authorities of every denomination, and every where retreating before the bayonet and the badge of office"—circumstances which, Mr. Tully justly conceives, distinguish it from miasmal or atmospheric diseases. As additional proofs of his position, he alludes to the well known fact, that merchant ships from Alexandria, Smyrna, and Constantinople, with their crews labouring under plague, have repeatedly, within the last three years, entered almost every port in the Mediterranean where the British flag was flying, (and many such instances took place during the author's residence in the Ionian Isles,) without being productive even of alarm, much less of danger—ships under such circumstances remaining for days in strict quarantine, and closely surrounded by guard-boats.

"Plague, to keep it out of a country, although at the very threshold, requires that it should only be treated as plague, subjecting it to the common rules of quarantine, and that upon the plain and simple principle of alone considering it what it truly is, contagious, and alone contagious." 223.

The author naturally slides to the subject of quarantine, on which he makes many important remarks. Experience, he observes, sufficiently proves—

"That susceptible effects, of whatever texture, and however impregnated with pestilential virus, can be securely purified by subjecting them to the combined, or even individual action of pure air, and water; and the more readily, when immersion is followed up by exposure to the all-powerful influence of certain degrees of heat. This was ascertained by actual proofs, both at Corfu and Cephalonia, but more especially at the latter island; and the period necessary for the purification of contaminated goods was found to be extremely limited under these processes. At Cephalonia, the tents which had been employed in our plague camps, after the simple process of being washed half a dozen times in salt water, and dried in the sun, were subsequently (with the most perfect conviction of the efficacy of the means of purification) delivered by me into His Majesty's stores, and soon after employed in the encampment of the garrison. Previous to our being thoroughly satisfied, that this process would prove effectual, many articles of this descriptien, from a suspicion of danger, had been destroyed. Our first trials originated in necessity, and, from the success that followed, we felt ourselves authorized to pursue those trials, which terminated as favourably as we could possibly wish." 229.

From this it would appear that every bale of goods received into a public lazaretto may, with proper attention, be quickly freed from all infectious properties—and that, if danger does exist, it will speedily declare itself.

"This admitted, we are perfectly authorized to conclude, that, under strict and efficient means, the actual state of the most extensive cargoes of susceptible goods, can be ascertained within fourteen days." 230.

At all events, he thinks that a quarantine expurgation extended to 21 days, would be perfectly efficacious, "under the most suspicious circumstances."

Our limits have already been considerably overleaped, and therefore we shall notice but one more subject, namely, the question whether man be really susceptible of repeated attacks of plague? Although Mr. Tully is not prepared with proofs sufficient to disprove satisfactorily the susceptibility of the human constitution to second attacks; yet his own conviction, from what he has seen and learnt, is, that no such secondary attacks take place. Thus of twelve persons employed as expurgators and hospital-attendants at Corfu, ten had the plague at Malta three years previously, and the other two, at Constantinople. All these continued free from the disease throughout the visitation at Corfu.

"During this exemption from sickness on the part of these persons, all our best exertions to secure their fellow labourers who had never had the disease, too frequently proved abortive; and it is deserving notice, that the only individual amongst the corps of expurgators sent from Malta, who had not previously had the disease, early fell a victim to it at Corfu." 237.

We are unable to enter into the 10th chapter of the work, or the documents appended thereto, though they will be perused with advantage by all those interested in the question at issue. We give Mr. Tully great credit for his zeal and ability in this investigation, and although we doubt some of his premises, we think him right, on the whole, in his conclusions. We fully coincide in the propriety of his measures for preventing or suppressing the contagion of plague, when it unfortunately appears, and we part with him entertaining both respect and esteem for his professional abilities and humane character.

---

When we had finished Mr. Tully's work, the elaborate researches of Dr. Hancock were put into our hands, and like Sisyphus we had to roll the stone once more to the summit of the hill! We have perused Dr. Hancock's work, and we accord to him the meed of applause for candour, zeal, research, and ingenuity. That it would be totally impossible to give a proper analysis of his work, will be acknowledged by all who examine it, and for the following reasons stated by the author himself.

> "Let him (the reader) also notice, that the several parts have a bearing, more or less distinct, upon the general principle; and that the subject itself is vast in its extent, and comprehensive in its relation; so that a view of a single part cannot give a correct idea of the whole, nor ought the whole to be viewed but in connexion with every part." Pref. p. ix.

We shall probably give some account of the scope and tendency of Dr. Hancock's work on another occasion.

---

